# Influenza H5 Hemagglutinin DNA Primes the Antibody Response Elicited by the Live Attenuated Influenza A/Vietnam/1203/2004 Vaccine in Ferrets

**DOI:** 10.1371/journal.pone.0021942

**Published:** 2011-07-08

**Authors:** Amorsolo L. Suguitan, Xing Cheng, Weijia Wang, Shixia Wang, Hong Jin, Shan Lu

**Affiliations:** 1 MedImmune, Mountain View, California, United States of America; 2 Department of Medicine, University of Massachusetts Medical School, Worcester, Massachusetts, United States of America; University of Georgia, United States of America

## Abstract

Priming immunization plays a key role in protecting individuals or populations to influenza viruses that are novel to humans. To identify the most promising vaccine priming strategy, we have evaluated different prime-boost regimens using inactivated, DNA and live attenuated vaccines in ferrets. Live attenuated influenza A/Vietnam/1203/2004 (H5N1) candidate vaccine (LAIV, VN04 *ca*) primed ferrets efficiently while inactivated H5N1 vaccine could not prime the immune response in seronegative ferrets unless an adjuvant was used. However, the H5 HA DNA vaccine alone was as successful as an adjuvanted inactivated VN04 vaccine in priming the immune response to VN04 *ca* virus. The serum antibody titers of ferrets primed with H5 HA DNA followed by intranasal vaccination of VN04 *ca* virus were comparable to that induced by two doses of VN04 *ca* virus. Both LAIV-LAIV and DNA-LAIV vaccine regimens could induce antibody responses that cross-neutralized antigenically distinct H5N1 virus isolates including A/HongKong/213/2003 (HK03) and prevented nasal infection of HK03 vaccine virus. Thus, H5 HA DNA vaccination may offer an alternative option for pandemic preparedness.

## Introduction

Influenza pandemics of varying severities have occurred in the last century. The 1918 “Spanish flu” (H1N1) that claimed the lives of about 40 million people is perhaps one of the deadliest pandemics in history [1]. The 1957 “Asian flu” (H2N2) and the 1968 “Hong Kong flu” (H3N2) pandemics are believed to have been caused by viruses from reassortment of circulating human strains and avian viruses [2]. The recent emergence of the swine-origin influenza A H1N1 pandemic (pH1N1) virus is a sober reminder that viruses with novel antigenic properties can infect and spread among an immunologically naïve human population with potentially devastating consequences.

Among the avian influenza viruses that have sporadically infected humans, highly pathogenic avian influenza (HPAI) H5N1 viruses pose the greatest threat due to their high virulence. As of February 2011, there have been 525 laboratory-confirmed cases of H5N1 infection, resulting in 310 deaths (∼59% mortality) [3]. There are concerns that H5N1 viruses could evolve and adapt to replicate and spread in the human population or gain human-to-human transmissibility through reassortment with circulating human influenza A viruses [4]. The 2009 pH1N1 virus has a high genetic compatibility with an avian H5N1 virus, raising the possibility that HPAI H5N1 viruses could acquire the ability of being readily transmitted among individuals [5]. Thus, the development of safe and efficacious vaccines against these viruses is a public health priority.

Vaccination is an integral component of strategies aiming to prevent and control pandemic influenza. Designed to mimic the route of natural infection, live attenuated influenza virus (LAIV) vaccines induce both local mucosal and systemic immunity [6] and are able to elicit broad immune responses against antigenically drifted strains [7], [8], [9], [10]. An H5N1 LAIV vaccine was generated by reverse genetics by combining the surface glycoprotein gene segments of A/Vietnam/1203/2004 (H5N1, VN04) and the six internal protein gene segments of the cold-adapted A/Ann Arbor/6/60 (H2N2, AA *ca*) master donor vaccine strain that confer the cold-adapted (*ca*), temperature-sensitive (*ts*), and attenuation (*att*) phenotypes to the reassortant vaccine virus [11], [12]. Our previous pre-clinical studies showed that a single dose of VN04 *ca* elicited low levels of neutralizing antibodies in mice and ferrets four weeks after immunization. Although a single dose of VN04 *ca* completely protected animals from challenge infection of lethal doses of homologous and heterologous H5N1 wild-type (*wt*) viruses, two doses of VN04 *ca* were required for complete protection from pulmonary virus replication [12].

To prevent or control influenza pandemics caused by HPAI H5N1 strains, multiple vaccinations or different vaccine prime boost approaches might be needed. DNA vaccination with plasmids expressing influenza viral proteins from the highly variable hemagglutinin (HA) to the more conserved matrix and nucleoprotein have been shown to induce humoral and cell-mediated immune responses in various animal species [13], [14], [15]. Although DNA vaccination can induce antibody responses comparable to unadjuvanted protein antigens [16], DNA vaccine alone is not as efficient as an adjuvanted protein vaccine. However, DNA vaccines could serve as a priming agent to significantly increase the immunogenicity of a protein vaccine. Such DNA prime-protein boost approach has been successfully exploited to improve the breadth of the cellular and humoral immune response elicited by various vaccines against different bacterial and protozoan pathogens in animal studies [17], [18], [19], [20], [21], as well as in an HIV vaccine study in humans [22]. Wei et al. (2010) recently reported that H1 HA DNA priming followed by a TIV boost not only led to increased neutralizing antibody titers but also broadened the response to antigenically distant H1N1 virus strains [23]. Huber et al. (2009) showed that boosting H3 HA DNA-primed mice with H3N2 and PR8 reassortant viruses induced a robust and broad antibody response against multiple H3N2 virus strains [24].

Since LAIV vaccination encourages development of a durable mucosal immune response and robust cell-mediated immunity, we evaluated several heterologous prime-boost regimens that would augment the immunogenicity of live attenuated VN04 *ca* candidate vaccine in ferrets. Our results indicate that an H5 HA DNA vaccine successfully primed the immune response that was subsequently boosted by VN04 *ca* virus. The protective immune response induced by H5 HA DNA prime-VN04 *ca* virus boost is comparable to that elicited by two doses of VN04 *ca* virus; both regimens protected ferrets from an antigenically distinct H5N1 virus replication in the respiratory tract.

## Results

### Antibody response from LAIV and inactivated H5N1 protein vaccine regimen

An unadjuvanted inactivated VN04 (iVN04) monovalent subvirion influenza vaccine was previously shown to be less immunogenic in humans and requires a vaccine dose of 90 µg of HA antigen in multiple doses to induce an antibody response similar to that of seasonal influenza vaccine [25], [26]. As vaccine supply will likely be limited during a pandemic, administering such large quantities of antigen in multiple doses would be unrealistic and inefficient. We therefore compared various prime-boost strategies in seronegative ferrets using iVN04 and VN04 *ca* virus to determine whether priming with either vaccine will be more immunogenic than immunization with a single form of the vaccine (Table 1). Ferrets primed with iVN04 did not have any detectable serum neutralizing antibody titers (Nt Ab) 28 days post-vaccination (Groups 2–4), but all ferrets primed with VN04 *ca* virus had detectable levels of serum Nt Ab after the first dose (Groups 5–7). The serum Nt Ab titers (51–62) elicited by a single dose of VN04 *ca* virus reported in this study are slightly higher than what was reported previously [12]. The Nt Ab titers in ferrets that received only a single dose of VN04 *ca* virus declined from 51 to 20 at 8 weeks post-vaccination (Group 5). Vaccination of ferrets with iVN04 adjuvanted with TiterMax, a stable water-in-oil emulsion commonly used in animal studies, elicited serum Nt Ab response (titer of 48) after the first dose and further increased to a titer of 190 following a boost with VN04 *ca* virus (Group 8). Two doses of iVN04 failed to induce any serum Nt Ab response (Group 3) due to lack of live virus priming in seronegative hosts [27]. Boosting of animals primed with iVN04 (Group 4), VN04 *ca* virus (Group 6), or adjuvanted iVN04 (Group 8) induced higher serum Nt Ab titers than that elicited by a single dose. However, iVN04 is the least efficient in priming the antibody response (titer of 95 vs 160 or 190). The antibody response of VN04 *ca* virus-primed ferrets was boosted with iVN04 without adujvant (Group 6). Due to the small sample size of animals used in this study (n = 4/group), statistically significant differences in the geometric mean antibody titers between different groups were not consistently achieved. Despite this caveat, our data indicated that VN04 *ca* virus or adjuvanted iVN04 vaccine can successfully prime seronegative hosts to mount a higher immune response following boost with VN04 *ca* virus or iVN04.

**Table 1:** Ferret serum antibody titers from vaccination with iVN04 and VN04 *ca*.

Group	Vaccine dose		GMT Nt antibody against VN04 *ca* virus	
	Prime	Boost	Post dose 1	Post dose 2
1	PBS	PBS	<10	<10
2	iVN04	PBS	<10	<10
3	iVN04	iVN04	<10	<10
4	iVN04	VN04 *ca*	<10	95
5	VN04 *ca*	PBS	51	20
6	VN04 *ca*	iVN04	62	160
7	VN04 *ca*	VN04 *ca*	62	123
8	iVN04/TiterMax	VN04 *ca*	48	190

Ferrets were inoculated with 10^7^ PFU of VN04 *ca* intranasally or 15 µg of iVN04 vaccine intramuscularly. Blood was collected 4 weeks after each dose and the H5N1-specific antibody levels (expressed as geometric mean titers) were determined by microneutralization assay.

### Expression of H5 HA protein from DNA

**Figure 1:**
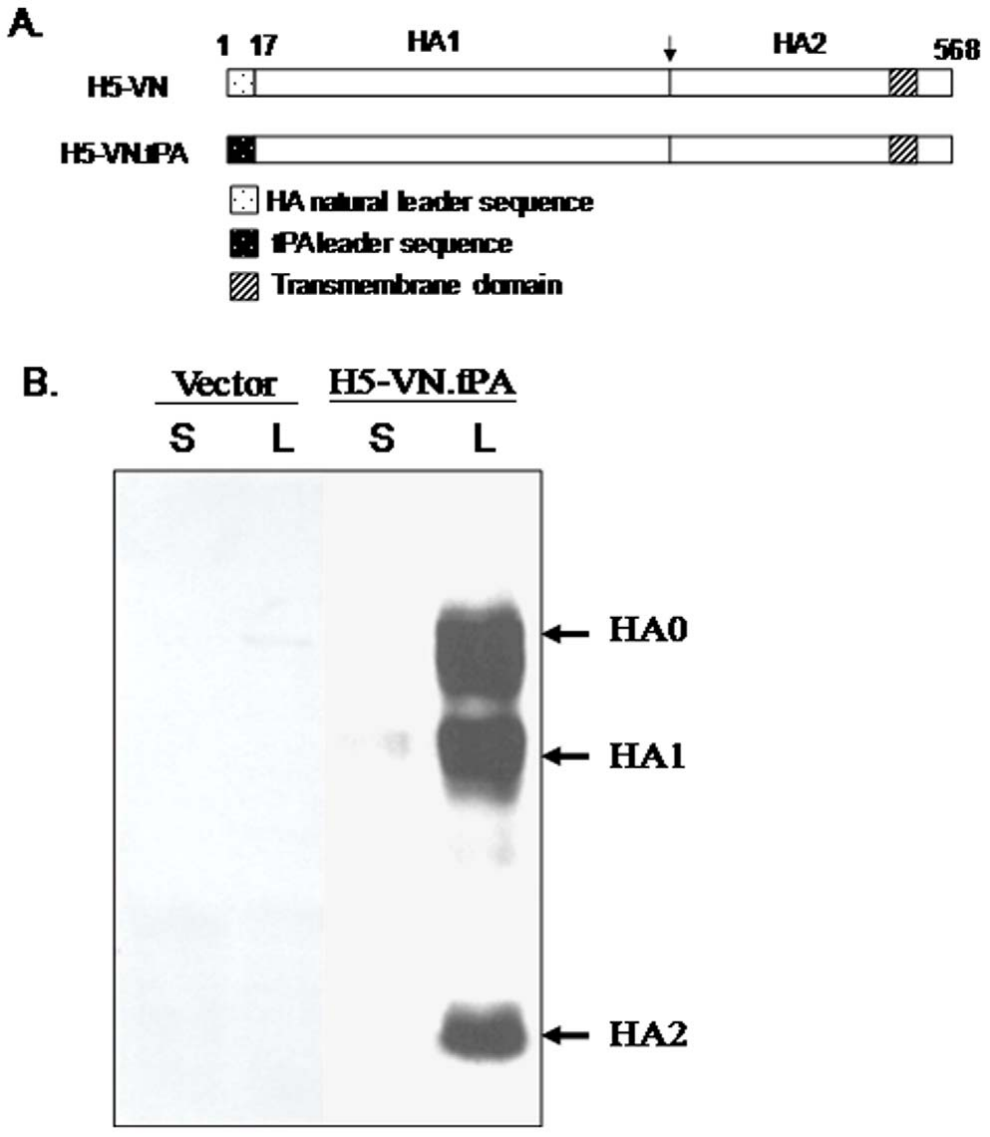
H5 HA DNA vaccine construct. (A) Schematic diagram of H5-VN.tPA HA gene. The H5-VN.tPA DNA encodes the full length HA antigens with a human tissue plasminogen activator (tPA) leader substituting for the natural HA leader sequence. The cleavage site between HA1 and HA2 subunits are indicated. The numbers above the HA inserts denote the relevant amino acid positions in natural HA proteins. (B) Western-blot analysis of the expression of HA antigen by H5-VN.tPA DNA vaccine in transiently transfected 293T cell supernatant (S) and cell lysate (L).

Since the use of a pandemic LAIV such as VN04 *ca* vaccine virus prior to a pandemic may be restricted and iVN04 requires adjuvant or a larger dose to prime the immune response against novel influenza viruses, the prime-boost regimen of H5 HA DNA and VN04 *ca* virus was thus considered for evaluation in ferrets. Our previous studies showed that the HA antigens were expressed more efficiently when the natural leader sequence was replaced with the tPA leader sequence [28]. Thus, the H5 VN tPA DNA containing tPA and codon-optimized HA cDNA including the ectodomain, transmembrane domain and cytoplasmic tail (Figure 1A) was used in the current study. The expression of H5 HA antigen from H5 DNA-transfected 293T cells was confirmed by Western blot (Figure 1B). The HA protein remained cell-associated; no protein was detected in the transfected cell culture supernatant. The H5 HA DNA vaccine contains multi-basic amino acid sequences at the cleavage site between the HA1 and HA2 (PQREXRRKKR↓G). The expressed HA protein was cleaved to HA1 and HA2 in addition to the HA0 in the transfected cells.

### Antibody response from H5 HA DNA prime and H5N1 LAIV boost regimen

**Figure 2:**
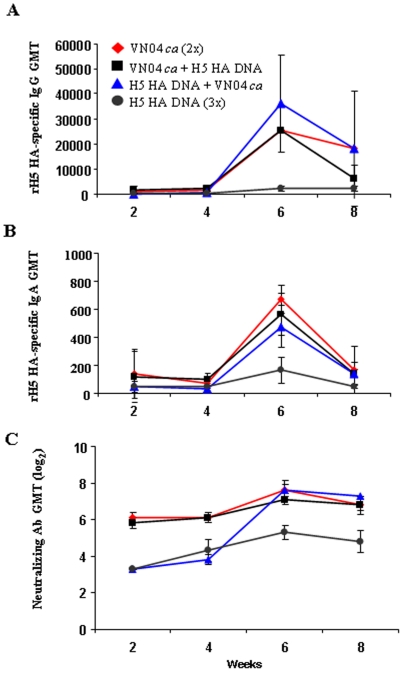
Kinetics of the development of H5 HA-specific serum IgG (A), IgA (B), and neutralizing antibodies (C) elicited by various H5N1 prime-boost regimens. Ferrets were immunized with either 10^7^ PFU of VN04 *ca* virus (weeks 0 and 4) or 200 µg of H5 HA DNA (weeks 0, 2 and/or 4) and serum samples were collected regularly at 2-week intervals. H5 HA-specific IgG and IgA in ferret serum samples (N = 4/group) were determined by ELISA using recombinant VN04 H5 HA as antigen. Neutralizing antibody titers against 100 TCID_50_ of the VN04 *ca* virus are depicted. Geometric mean titers from each group are shown.

Serum antibody responses were measured by ELISA, HAI and neutralization assays. Recombinant H5 HA-specific IgG and IgA antibody titers were determined and presented in Figure 2. Very low levels of HA-specific IgG antibody were detected in ferrets that received the H5 HA DNA vaccine alone, even after the third dose (Figure 2A) when the needle injection was used to deliver the DNA vaccine. In contrast, ferrets that were primed with the H5 HA DNA and boosted with VN04 *ca* virus reached a slightly higher mean HA-specific IgG titer than ferrets that received two doses of VN04 *ca* virus or VN04 *ca* virus prime-H5 HA DNA boost (Figure 2A). These three vaccine regimens also elicited comparable levels of HA-specific IgA serum titers while DNA vaccination alone induced very modest levels of HA-specific IgA antibody (Figure 2B). Interestingly, both the H5 HA-specific serum IgG and IgA titers elicited by the various vaccine regimens declined four weeks after the boost. This decrease in influenza-specific antibody titers has also been described previously for a seasonal H3N2 strain in mice [29]. In contrast to binding antibodies, the serum Nt Ab titers remained at comparable levels between two and four weeks after the boost (Figure 2C).

**Figure 3:**
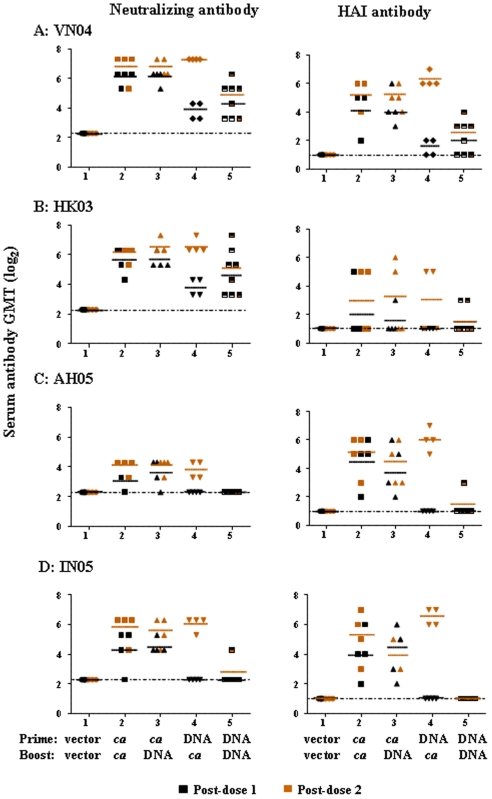
Serum antibody titers against homologous and variant H5N1 *ca* viruses following different vaccine regimens in ferrets. Ferrets (N = 4/group) were inoculated with 10^7^ PFU of VN04 H5N1 *ca* intranasally or 200 µg of vector or H5 HA DNA vaccine intramuscularly as indicated. Blood was collected 4 weeks after each dose and H5N1-specific antibody titers in the serum were determined by microneutralization (left panel) and HAI (right panel) assays. Geometric mean titers of neutralizing and HAI Ab against (A) VN04 (A/Vietnam/1203/2004), (B) HK03 (A/Hong Kong/213/2003), (C) AH05 (A/Anhui/1/2005), and (D) IN05 (A/Indonesia/5/2005) *ca* viruses are depicted. Data points in black indicate titers of post-prime and data points in orange indicate titers of post-boost and the mean Ab titers are indicated in horizontal solid lines. The dashed line indicates the detection limit of the assays.

The levels of HA-specific antibodies in immunized ferret sera were also measured by microneutralization assay (left panel) and HAI assay (right panel, Figure 3). The breadth of the serum Nt and HAI Ab titers against antigenically distinct H5N1 influenza A/Hong Kong/213/2003 from clade 1 (HK03), A/Anhui/1/2005 from clade 2.3.4 (AH05), and A/Indonesia/5/2005 from clade 2.1 (IN05) *ca* strains was also examined. Ferrets that were primed with VN04 *ca* virus displayed low levels of serum Nt Ab titers against the homologous vaccine virus 4 weeks post-immunization (Groups 2 and 3, Figure 3A, left panel) while ferrets that were primed with the H5 HA DNA exhibited barely detectable serum Nt Ab at the same time point (Groups 4 and 5). Ferrets that received 2 doses of VN04 *ca* virus (Group 2) or those that received the prime-boost regimen (Groups 3 and 4) had higher serum Nt Ab titers. Three doses of the H5 HA DNA vaccine elicited a low level of serum Nt Ab titers (Group 5). A very similar pattern of results was also observed for serum HAI Ab against the VN04 *ca* virus (Figure 3A, right panel).

The prime-boost strategies (Groups 3 and 4, Figure 3) induced serum Nt and HAI Ab titers that cross reacted to the variant H5N1 viruses tested in this study at levels comparable to that elicited by two doses of VN04 *ca* virus (Group 2). In contrast, ferrets that were immunized with the H5 HA DNA vaccine alone exhibited barely detectable Ab titers against AH05 and IN05 and low serum Nt Ab titers to HK03 (Group 5, Figure 3C, 3D and 3B, respectively).

**Figure 4:**
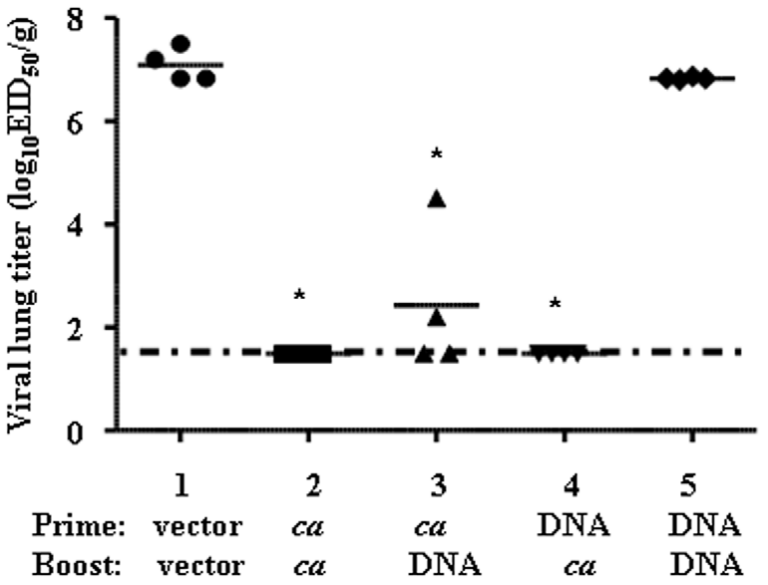
Protection conferred by different H5 vaccine prime-boost regimens in ferrets. Four weeks after boost immunization, ferrets were intranasally infected with 10^7^ PFU of HK03 *ca* virus. Nasal turbinates were collected on day 3 post-infection and viral titers in tissue homogenates were determined by EID_50_. Horizontal lines depict the mean virus titers for each group. An asterisk above a horizontal bar denotes statistical significance compared to the vector DNA group (*p*<0.05, Mann-Whitney U Test).

We then examined whether this cross-reactive antibody response to variant H5N1 viruses would confer protection in vivo (Figure 4). All immunized ferrets were challenged with the HK03 *ca* vaccine virus. Because of the attenuated phenotype of this challenge virus, viral replication could only be measured in the upper respiratory tract of ferrets. Ferrets that either received two doses of the VN04 *ca* virus or those that were primed with H5 HA DNA followed by VN04 *ca* virus boost were completely protected from HK03 *ca* virus replication in the nasal turbinates (Groups 2 and 4, *p*<0.05). The group that was primed with VN04 *ca* virus and boosted with H5 HA DNA also had significantly reduced viral titers in the upper respiratory tract (Group 3, *p* <0.05). The ferrets that received H5 HA DNA vaccine alone (Group 5) were not protected against HK03 *ca* virus replication; viral titer in the NT was similar to the vector control group (Group 1). Thus, similar to two doses of VN04 *ca* vaccination, H5 HA DNA prime-VN04 *ca* virus boost induced immune response that could provide protection against homologous and heterologous H5N1 virus.

## Discussion

The recent experience with the novel swine-origin 2009 H1N1 virus highlighted the importance of early, swift delivery and broad administration of vaccines during a pandemic. In the case of pH1N1 vaccine, a single dose without an adjuvant was highly immunogenic in adults and induced ∼90% seroprotective conversion rate [30], [31]. This is most likely due to the pre-existing cross-reactive antibodies to pH1N1 present in humans, especially the older adults who were previously exposed to seasonal H1N1 viruses [31], [32]. However, many populations lack any pre-existing immunity against the novel H5N1 viruses that have sporadically infected humans. Thus, priming might be an important strategy to reduce the mortality and morbidity caused by the HPAI H5N1 viruses.

Unlike the 2009 H1N1 virus that has binding specificity for both α2,6-linked (human-like) and α2,3-linked (avian-like) sialic acid (SA) receptors [33], [34], the H5N1 VN04 *ca* virus has binding preference for the α2,3-linked SA receptor [35]. The receptor binding preference of the H5N1 viruses may contribute to their inefficient transmission among humans and the low seroprevalence to this virus subtype among presumably exposed populations [36], [37], [38]. In addition, the glycosylation of the HA protein at position 158 also contributed to the low immunogenicity of VN04 *ca* [35]. Thus, priming susceptible populations with a pre-pandemic H5 vaccine would induce some cross-protective antibodies that could significantly augment the immunogenicity of an antigenically matched pandemic vaccine once it becomes available. The rapid immune response elicited by the pandemic vaccine, acting as a boosting agent, is expected to reduce the morbidity, mortality and transmission of the pandemic virus [33] and may decrease the number of doses required to achieve protective immunity.

In this study, the immunogenicity of the different vaccine regimens was assessed primarily by measuring HAI and Nt Ab in the serum of immunized ferrets. Although it is well established that LAIVs induce a robust mucosal immune response, we were unable to detect H5 HA-specific IgG and IgA antibody titers in the nasal wash due to assay insensitivity using the existing available ferret reagents in the ELISA assay. Two doses of iVN04 were not immunogenic in seronegative ferrets, consistent with the findings that purified protein antigens are not immunogenic in naïve animals [27]. However, priming with an adjuvanted iVN04 or VN04 *ca* virus could elicit high serum Nt Ab titers after the boost by either iVN04 or VN04 *ca* virus. These findings are in agreement with previous reports that adjuvants significantly improve the immunogenicity of inactivated H5N1 vaccines [39], [40]. However, adjuvanted influenza vaccines are currently not licensed for use in the United States. Concerns over possible genetic reassortment with circulating influenza virus strains also restrict the use of an H5N1 LAIV for priming. Therefore, an alternative priming strategy is needed for future pandemics that are caused by novel subtypes. We found that a codon-optimized H5 HA DNA vaccine was comparable to an adjuvanted iVN04 as a priming agent. H5 HA DNA vaccination was not effective when administered alone by needle injection, inducing serum Nt or HAI Ab at levels below the detection limits. Our previous experience indicated that DNA immunization by intramuscular injection was not robust; thus, an additional DNA immunization was administered 2 weeks after the first dose in this study. This extra DNA dose did not appear to enhance DNA vaccine-mediated antibody response. However, the H5 HA DNA vaccine can prime the immune system to rapidly mount an immune response after boosting with VN04 *ca* virus vaccine, reaching serum Nt Ab titers comparable to that elicited by two doses of the live attenuated VN04 *ca* vaccine. The H5 HA DNA vaccine is an attractive alternative over an adjuvanted iVN04 because the DNA can be produced quickly, is relatively inexpensive to manufacture, and is amenable to long-term stockpiling.

H5N1 viruses continue to diversify and evolve in wild birds and domestic fowl [41]. It is highly desirable to produce a vaccine that is broadly cross-reactive to diverse strains. Ferrets that received 2 doses of VN04 *ca* virus or the H5 HA DNA prime-VN04 *ca* virus boost regimen had serum Nt and HAI Ab responses against antigenically distinct H5N1 viruses from different clades and prevented replication of a heterologous HK03 *ca* vaccine virus. Our previous results indicate that mice immunized with a single dose of the VN04 *ca* vaccine were protected from various lethal doses of heterologous H5N1 *wt* viruses even in the absence of detectable Nt Ab in the serum, through a yet undefined immune mechanism [12]. Thus, the correlation between serum HAI or Nt Ab titers and protection from either lethal virus challenge or even virus replication in the respiratory tract of animals for H5N1 viruses is not firmly established. In this study, the ferrets that received the VN04 *ca* vaccine (whether as a prime or as a boost) with detectable serum Nt and HAI Ab against antigenically diverse H5N1 viruses are expected to survive a lethal HPAI H5N1 virus challenge based on our previous report [12]. It was recently reported that priming with H1 HA DNA is associated with the induction of a broader Nt Ab response to temporally distant H1N1 strains and these Nt Ab target the conserved HA stem region [23]. Sera from ferrets that received either two doses of VN04 *ca* virus or the H5 HA DNA prime-VN04 *ca* virus boost regimen also recognized the HA2 region of a recombinant H5 HA and the H1 HA protein of a seasonal H1N1 virus, A/South Dakota/6/2007, by Western blotting (data not shown). We were not able to demonstrate clear broader reactivity against H5N1 subtypes of the animals primed with DNA followed by boost with the VN04 *ca* virus. Even though H5 HA DNA vaccination elicited these HA2-binding antibodies, they were not sufficient to protect ferrets from replication of a heterologous H5N1 virus. Additional studies are required to determine the contributions of the stalk region-mediated response to subtypic and heterosubtypic immunity. It will also be important to understand the mechanism of protection induced by DNA vaccination and the contribution of cell-mediated immune responses to this protection.

Several strategies could be considered to further broaden the immune response elicited by H5 HA DNA priming by using heterologous H5 HA from genetically distinct clades or with multiple H5 HA DNA plasmids from representative H5N1 clades followed by boosting with the antigenically matched pandemic LAIV vaccine strain once it becomes available. A robust immune response towards the boosting strain can be achieved even when the doses are administered months or years apart [42], [43], [44]. A similar study is warranted to determine the durability of an H5 HA DNA priming so that the timing of additional doses could be planned. While the duration of protection conferred by the pandemic LAIV boost is not known, recent studies in children immunized with the seasonal trivalent LAIV show that relative efficacies against antigenically matched and mismatched strains can last more than 12 months post-vaccination [45], [46]. Another option to improve the immunogenicity of H5 HA DNA priming is to adopt a more efficient gene delivery method such as the use of a gene gun or electroporation [47]. However, the accessibility, affordability, safety, and ease of operating such devices for DNA vaccine delivery may limit their use, especially in a pandemic setting [48]. In the current study, DNA priming with conventional needle injection was proven effective. However, we only evaluated H5N1 vaccines in ferrets. The effectiveness of the DNA priming effect remains to be demonstrated for other influenza virus subtypes in pre-clinical studies; its safety and efficacy should be further evaluated in clinical studies.

## Materials and Methods

### Generation of recombinant vaccine viruses

Recombinant influenza VN04, HK03, IN05 and AH05 *ca* viruses with multi-basic amino acids removed from the H5 HA cleavage site were generated as described previously [12]. Recombinant 6:2 viruses bearing the modified HA and unmodified NA from each H5N1 virus along with the six internal protein gene segments of AA *ca* were rescued using the eight-plasmid transfection system [11], [49]. Viruses were amplified in the allantoic cavity of 10–11 day old embryonated chicken eggs. Viral titer was determined by plaque assay or 50% tissue culture infectious dose (TCID_50_) in Madin-Darby canine kidney (MDCK) cells.

### Codon-optimized H5 HA DNA vaccines

The codon optimized H5-VN.tPA DNA expressing the full length HA0 was cloned into DNA vaccine vector pSW3891 under a human tissue plasminogen activator (tPA) leader sequence. The DNA vaccine plasmid was prepared from *Escherichia coli* (HB101 strain) with a Mega purification kit (Qiagen, Valencia, CA) for both *in vitro* transfection and *in vivo* animal immunization studies. The HA protein expression was examined by transient transfection of 293T cells and Western blot analysis as previously described [28].

### Ferret studies

Eight- to ten-week old ferrets from Simonsen (Gilroy, CA) that were seronegative for antibodies to seasonal H1N1, H3N2 and H5N1 *ca* viruses were used in the studies. All animals were anesthetized with isoflurane prior to immunization. In the first experiment, 8 groups of 4 ferrets were primed with bilateral intra-muscular (i.m.) injections of one of the following: (1) PBS; (2) 15 µg of iVN04 (NR-4143, Biodefense and Emerging Infections Research Resources Repository, NIAID, NIH, Bethesda, MD); (3) 15 µg of iVN04 with the adjuvant TiterMax (TiterMax, USA); or (4) intra-nasally (i.n.) with 10^7^ PFU of VN04 *ca* virus. Four weeks after the first dose, ferrets were immunized with PBS, iVN04 or VN04 *ca* virus. H5 HA-specific antibody responses were evaluated using sera collected on days 28 and 56 post-vaccination.

In the second experiment, 5 groups of 4 ferrets were vaccinated with bilateral i.m. injections of one of the following: (1) 200 µg of plasmid vector (Group 1); (2) i.n. inoculation of 10^7^ PFU of VN04 *ca* virus (Groups 2–3) or (3) codon-optimized H5 HA DNA plasmid (Groups 4–5). Two weeks after priming, a group of ferrets that were primed with the H5 HA DNA vaccine received a second dose of the H5 HA DNA vaccine. Four weeks after the first dose, ferrets were immunized with PBS, H5 HA DNA or VN04 *ca* virus. Four weeks after the boost, all ferrets were i.n.-infected with 10^7^ PFU of HK03 *ca* virus. Ferrets were sacrificed on day 3 post-infection, the nasal turbinates were harvested and virus titers in the upper respiratory tract of ferrets was determined by 50% egg infectious dose (EID_50_) assay and expressed as log_10_ EID_50_/g of tissue. H5 HA-specific antibody responses were evaluated using sera collected on days 14, 28, 42 and 56 post-vaccination. All animal studies were conducted in accordance with the Institutional Animal Care and Use Committee-approved protocols (058-07-035 and 081-09-053).

### HAI and microneutralization assays

Influenza-specific serum antibody responses against the homologous and heterologous H5N1 viruses of immunized ferrets were determined by microneutralization assay and HAI assay. Briefly, serum samples were treated with receptor-destroying enzyme (Denka-Seiken, Tokyo, Japan) at 37°C overnight followed by heat-inactivation at 56°C for 45 min. For HAI assay, serial 2-fold dilutions of serum were incubated with an equal volume of 4 HA units of the indicated H5N1 *ca* viruses in a 96-well V-bottom plate for 1 hr, followed by the addition of either 1.0% horse erythrocytes (Lampire Biological Laboratories, Pipersville, PA) containing 0.5% BSA (against VN04, AN05 and IN05) or 0.5% turkey erythrocytes (Lampire Biological Laboratories, Pipersville, PA) (against HK03). HAI antibody titers were defined as the reciprocal of the highest serum dilution that completely inhibited hemagglutination. For microneutralization assays, serial 2 fold-dilutions of serum were incubated with an equal volume of 100 TCID_50_ of the indicated H5N1 *ca* viruses at 33°C for 1 hr. The virus-serum mixture was then transferred to monolayers of MDCK cells and incubated at 33°C for 6 days. Neutralizing antibody titer was defined as the reciprocal of the highest dilution of serum that completely neutralized virus infectivity as indicated by the absence of cytopathic effects (CPE) on day 6 post-infection.

### ELISA

An enzyme-linked immunosorbent assay (ELISA) was conducted to measure H5 HA-specific IgG and IgA antibody titers in the sera of immunized ferrets. Half of a 96-well flat bottom plate (Costar, Corning, NY) were coated with 0.05 µg of recombinant VN04 H5 HA protein (Protein Sciences, Meriden, CT) and the other half of the plates were incubated with 100 µl of PBS. The plates were incubated at 4°C overnight, washed 3x with PBS containing 0.05% Tween 20 (Sigma, St. Louis, MO), and then blocked with 300 µl of blocking buffer (SuperBlock blocking buffer in PBS, ThermoScientific, Rockford, IL) for 1 hr at 37°C. After removing the blocking buffer from the plates, serial 4-fold dilutions of serum samples (in 10% SuperBlock blocking buffer + 0.05% Tween 20; for IgG assay) or serial 2-fold dilutions of serum samples (for IgA assay) were added to the wells with or without the coating antigen, and incubated for 1 hr at 37°C. After washing, the plates were incubated with 100 µl of 1∶10,000 dilution (in 10% SuperBlock + 0.05% Tween 20) of goat anti-ferret IgG conjugated to horse radish peroxidase (Bethyl Laboratories, Inc., Montgomery, TX) (for IgG assay) or 100 µl of 1∶2,500 dilution of goat anti-ferret IgA (Bethyl Laboratories, Inc., Montgomery, TX) conjugated to biotin (EZ-link Sulfo-NHS-SS biotinylation kit, ThermoScientific, Rockford, IL) (for IgA assay) for 45 min at 37°C. After washing, the IgA plates were further incubated with 1∶10,000 dilution of streptavidin-HRP (ThermoScientific, Rockford, IL) for 30 min at 37°C. Both IgG and IgA assay plates were developed with 100 µl of 3,3′,5,5′ tetramethylbenzidene (TMB) substrate solution (Sigma, St. Louis, MO); IgG plates were incubated for 5 min at room temperature while IgA plates were incubated for 30 min at 37°C. The reactions were stopped by adding 100 µl of 1 N H_2_SO_4_ and the absorbance were read at λ = 450 nm. The difference in the OD readings of each diluted sample between wells coated with or without the H5 HA antigen was determined, and the H5 HA-specific IgG and IgA titers were defined as the reciprocal of the highest dilution that gave a ΔOD value ≥ 0.200.
